# Differential impact of yeast cell wall products in recovery of porcine intestinal epithelial cell barrier function following Lipopolysaccharide challenge

**DOI:** 10.1186/s40813-023-00312-2

**Published:** 2023-04-17

**Authors:** Niall Browne, Daniel Daly, Karina Horgan

**Affiliations:** Alltech Bioscience Centre, Summerhill Road, Dunboyne, Co. Meath Ireland

**Keywords:** IPEC-J2, Intestinal epithelial barrier integrity, Tight junction proteins, Mannan rich fraction, Yeast cell wall products

## Abstract

**Background:**

In swine intestinal barrier deterioration can be caused by exposure to harmful bacteria, toxins or contaminants that can lead to a leaky gut and post weaning diarrhoea. A leaky gut leads to increased infection, inflammation and poor nutrient absorption that can impair piglet growth and ultimately survival. Application of yeast cell wall (YCW) products may offer an opportunity to reduce the intestinal barrier damage caused by microbial challenge. A Mannan rich fraction (MRF) and three YCW products were compared by examining their impact on intestinal barrier function using a Jejunal model of intestine in response to a bacterial challenge using *Salmonella* LPS.

**Results:**

Trans epithelial electrical resistance (TEER) readings showed MRF had a significantly higher barrier function (*P* ≤ 0.05) over the positive control while YCW products A, B and C demonstrated no significant improvement to the positive control. Transcriptome analysis of the IPEC-J2 cells showed that differentially expressed genes associated with the gene ontology (GO) term for Structural molecule activity was significantly upregulated in the MRF treated cells over the positive control cells with 56 genes upregulated compared to product B (50 genes), Product C, (25 genes) and the negative control’s 60 genes. Product A had no functional grouping under the structural molecule activity term.

Both qPCR and western blotting analysis of tight junction associated genes showed that MRF treated cells demonstrated significantly higher Claudin 3 junctional gene expression (*P* ≤ 0.05) over the positive control and treatments A, B and C. Occludin expression was significantly higher in MRF treated cells (*P* ≤ 0.05) over the positive control and product B. A nonsignificant rise in TJP-1 gene expression was observed in the MRF treated cells when compared to the positive control. Protein abundances of Claudin 3, Occludin and TJP-1 were significantly (*P* ≤ 0.05) higher following MRF application to LPS challenged IPEC-J2 cells over the positive control.

**Conclusions:**

The difference in each YCW products production and composition appeared to influence intestinal barrier integrity. The action of MRF demonstrates its potential ability to raise intestinal barrier integrity of IPEC-J2 intestinal cells on an in vitro level through significantly elevated intracellular connections.

**Supplementary Information:**

The online version contains supplementary material available at 10.1186/s40813-023-00312-2.

## Background

Intestinal barrier function is central to nutrient absorption and the physical regulation of pathogenic bacteria, harmful toxins and antigenic material from entering animals. The regulation and maintenance of the gut barrier is key to sustaining growth and maintenance of pigs and other animals by facilitating the selective transport of nutrients, water and electrolytes. The barrier’s selectivity is regulated by tight junctions including claudins, occludin and zonula occludens (tight junctional protein) that can mechanically compartmentalise the absorption of nutrients in tandem with selective ion transporters [1, 2].

Issues that affect the regulation of the barrier can give rise to a leaky gut particularly at weaning which is further affected from infections picked up during this susceptible period when passive immunity is declining. With commercial settings having a shorter weaning time of 14–30 days compared to what occurs in nature at 70–84 days. This shorter time frame in combination with weaning stresses and increased exposure to pathogens from mingling of piglets restricts the time for barrier integrity to develop [2]. Several studies have established strong links with weaning leading to breakdown of the intestinal barrier when assessed by trans epithelial electrical resistance [3, 4].

Main et al., [5] demonstrated that barrier integrity was improved with delayed weaning age from 12 to 21.5 days resulting in direct improvements in growth rate, feed efficiency and reduced mortality rates although still a shorter weaning period than observed in nature. Evidence from early weaning has also highlighted a continued impact into adulthood from early disruption of the intestinal tract development on barrier function and immune function [6, 7].

McLamb et al., [8] demonstrated that piglets weaned from 15 days with an *E. coli* challenge had increased diarrhoea and intestinal permeability, and poorer growth performance compared to pigs weaned at 22 days. Early weaning led to lower levels of IL-6 and IL-8 proinflammatory cytokines which are central to immune coordination and repair. The reduced barrier capacity can facilitate increased incidences of infection from *E. coli* and *Salmonella* species which can be transmitted to human consumers.

In addition to transmissibility of *Salmonella* to human consumers having potentially life-threatening consequences, the presence of *Salmonella* species in piglets can contribute to post weaning diarrhoea and poor overall performance due to a weakened intestinal barrier function.

Exposure to microbes also affects cell replacement rates of the intestinal tract which negatively impacts on both barrier function and the pigs growth [9]. Piglets are at a greater risk to pathogen colonisation during weaning [10, 11], this may offer *Salmonella* and *E. coli* species the ability to establish despite the use of non-antibiotic interventions such as zinc oxide. The introduction of a ban on the use of Zinc Oxide use at therapeutic levels above 150 ppm in 2022 for pig farmers in the European Union will compound the difficulty in controlling *Salmonella* species in pigs especially during weaning and an alternative means to limit *Salmonella* load in pigs is required by the industry. Alternatives that have been utilised include probiotics [12], acidifiers [13] and prebiotics such as Mannan oligosaccharides (MOS) [14, 15] with the latter assessed in this study in vitro with a mannan rich fraction (MRF) and yeast cell wall based products.

MOS products derived from *Saccharomyces cerevisiae* subvert the means by which pathogenic bacteria invade the host intestinal tract by binding to mannan molecules that coat the intestines [16]. Mannose carbohydrate binding specificity of the surface lectins of enteric bacteria such as *Salmonella typhimurium* was previously described by Firon et al., [17]. Most studies that use cell lines originated from intestinal epithelial cells, including IPEC-J2 [18], ICE-1 [19], or HT-29 [20], demonstrated type 1 fimbriae dependent binding of *Salmonella* to mannan molecules on the intestinal cells. The MRF used here structurally resembles the receptor sites of the intestinal epithelium which acts as a decoy receptor for pathogens like *Salmonella* to adhere to thus altering the level of infection [21].

Mannan from yeast has previously demonstrated its capacity to limit infection in animals susceptible to gastrointestinal infection including pigs [14, 15, 22, 23]and poultry [24–26] by blocking the mechanism by which gram-negative bacteria adhere to and invade the intestine.

The work outlined here assessed MRF and other YCW products’ effects on intestinal barrier function in response to a bacterial challenge from *Salmonella* lipopolysaccharide (LPS) (Table 1).

**Table 1:** List of the primers used for gene expression analysis by quantitative real-time PCR in IPEC-J2 cells

Gene	Primer	Accession
*Occludin*	F: 5’-GGTCTCTCTACAGCTCACAAATC, R: 5’-CCACCACGCAGTAGTGATATAAA	UP000008227
*Claudin-3*	F: 5’-GAGAGTCGTACACTTTGCACTG R: 5’-TCATCGGCAGCAGCATTATC	NP001153547.1
*TJP-1*	F: 5’-GAGAAGTGCCAGTAGGGATAAG, R: 5’-GTGTGACTTTGGTGGGTTTG	A0A287BF71
*GAPDH*	F: 5’-AAGGAGTAAGAGCCCCTGGA R: 5’-TCTGGGATGGAAACTGGAA	P00355

## Results

### Trans epithelial electrical resistance of IPEC-J2 cells challenged with LPS in the presence or absence of YCW

**Fig. 1:**
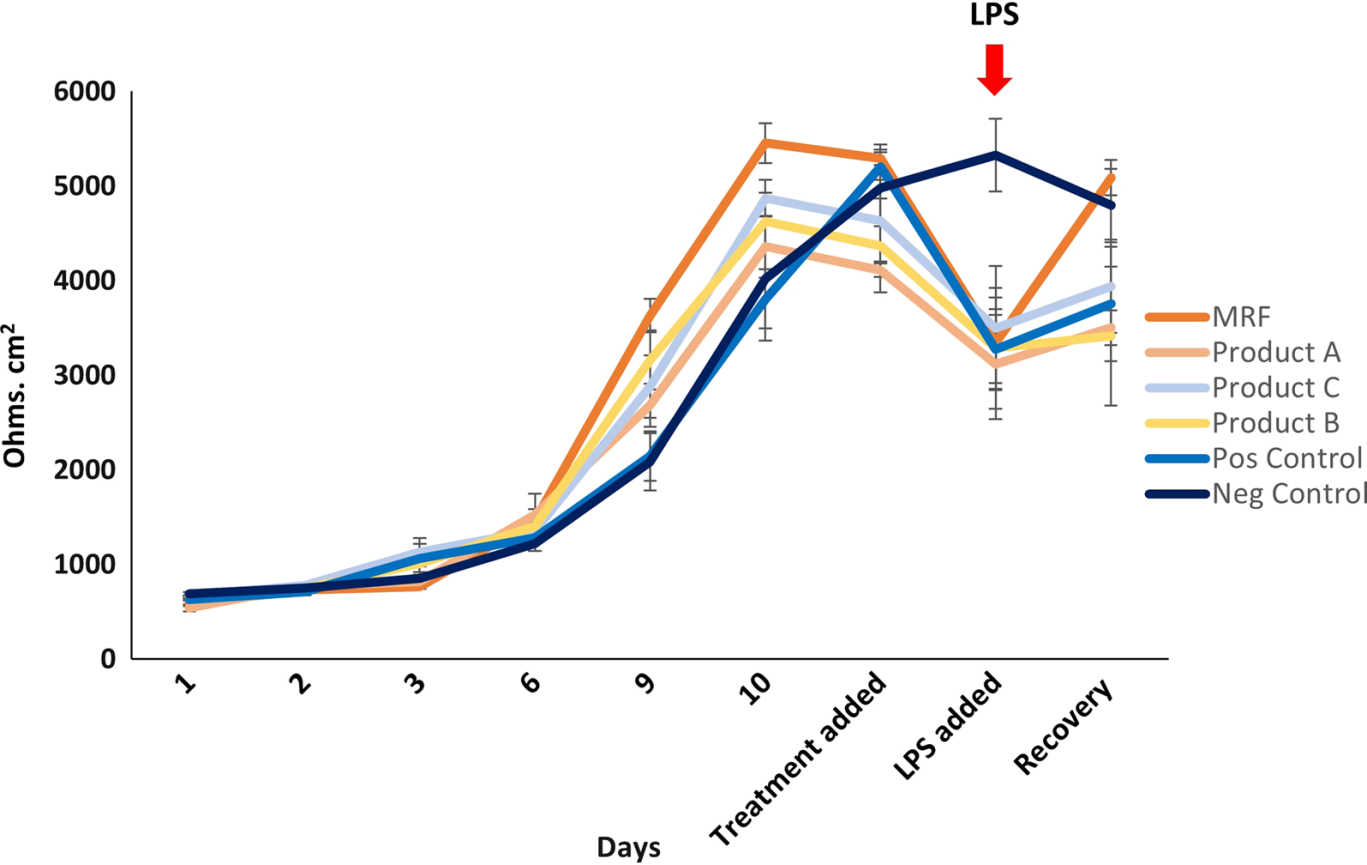
Trans epithelial electrical resistance (TEER) from differentiated IPEC-J2 cells challenged with LPS and treated with MRF, product A, C, or B and positive Control (Pos Control) and negative Control (Neg Control). Significance and non significance (ns) was marked in Table 2 (*n* = 4)

Differentiated IPEC-J2 cells were grown on 12 well inserts for 14 days followed by exposure to YCW products and an LPS challenge to examine the deterioration and recovery of the intestinal barrier. Cells receiving MRF demonstrated the greatest recovery in barrier function compared to the other product treatments (Fig. 1). Both the positive control with LPS (3754.2 ± 605.8, Ohms. cm^2^) and product B (3414.289 ± 733.9) had the lowest rate of recovery followed by product C (3938.4 ± 491.4) and product A (3502.5 ± 182.8). MRF (5090.3 ± 187.0, *P* ≤ 0.05) demonstrated a significantly greater degree of TEER barrier recovery over the positive control (+ LPS) (3754.2 ± 605.8) (Table 2).

**Table 2:** TEER readings (Ohms. cm^2^) of LPS challenged IPEC-J2 cells receiving MRF and YCW product applications compared to the Positive control (+ LPS)

	MRF v pos control	Product A v pos control	Product B v pos control	Product C v pos control	Neg control v pos control
Product application	ns	ns	ns	ns	ns
LPS Challenge	ns	(*P* ≤ 0.05) *	ns	(*P* ≤ 0.05) *	(*P* ≤ 0.05) *
Recovery	(*P* ≤ 0.05) *	ns	ns	ns	(*P* ≤ 0.05) *

### Venn diagram of differentially expressed genes

**Fig. 2:**
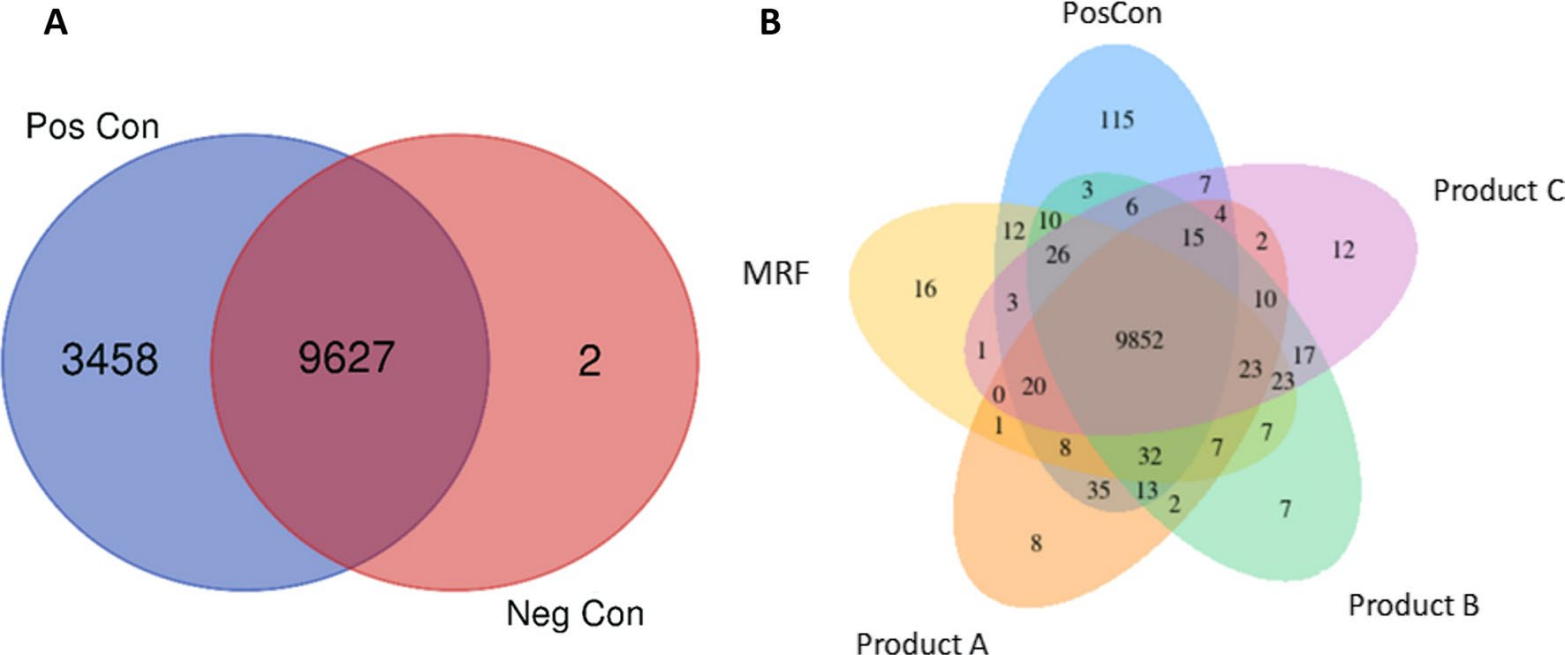
Venn diagram showing the number of differentially expressed genes, **A** Number of differentially expressed genes among IPEC-J2 cells challenged with LPS only in the positive control (PosCon) or negative control (NegCon) without LPS challenge. **B** Number of differentially expressed genes among IPEC-J2 cells challenged with LPS and treated with either MRF, product A, B or C compared to the positive control (PosCon), (*n* = 4)

Comparing the LPS exposed cells to non-exposed IPEC-J2 cells demonstrated 3458 genes were differentially expressed when cells were exposed to LPS, whereas only 2 genes were identified as unique to the untreated negative control (Fig. 2A). This demonstrates the validity of the LPS application to IPEC-J2 cells stimulating a strong differential gene expression response to the unchallenged negative control. Venn diagram of differentially expressed genes depicts differences and similarities between IPEC-J2 cells receiving YCW products and the positive control which demonstrated a large number of commonly expressed genes (9852) likely attributed to the fact they were all exposed to the same LPS challenge. Although the positive control had 115 uniquely distinct genes in comparison to all other treated groups, MRF had 16 genes, Product A, eight genes, Product B, seven genes and Product C, twelve genes (Fig. 2B).

### Differentially upregulated genes compared to the positive control

The top 5 upregulated gene transcription fold changes relative to the positive control for each treatment in response to LPS challenge or no challenge for the negative control were compiled (Table 3). Similar top 5 gene fold changes appeared in different treatments. A further list was generated for fold changes based on functions. Fold changes of two fold and above were deemed significant when compared to the positive control.

**Table 3:** The top 5 upregulated gene transcription fold changes relative to the positive control for each treatment in response to LPS challenge or no challenge for the Negative control were compiled

Positive control	MRF	Product A	Product B	Product C	Negative control	gene name	Description
1	15	0	5	6	0.166	CIB3	Integrin
1	13	5	4	4	1	CASS4	Cellular adhesion
1	11	12	3	18	0.2	TLL2	Degradation of the extracellular matrix and Collagen formation
1	2.666	2.666	15.66	18	0.375	SGCA	Muscle fiber membranes and to the linking of the actin cytoskeleton to the extracellular matrix
1	0.818	1.272	0.272	0.909	11	ARMC12	Mitochondrial peripheral membrane protein and functions as an adherence factor between mitochondria in cultured cells
1	8	2	13	7	0.071	IGFBP4	IGF-binding proteins prolong the half-life of the IGFs and have been shown to either inhibit or stimulate the growth promoting effects of the IGFs on cell culture
1	6	12.25	7.5	11.75	0.5	NR4A3	Regulation of proliferation, survival and differentiation of many different cell types and also in metabolism and inflammation
1	0.375	0.25	24	10.375	1.6	INHBA	This gene encodes a member of the TGF-beta (transforming growth factor-beta) superfamily of proteins
1	17	13	24	25	0.090	CLSTN3	Calcium-mediated postsynaptic signals
1	11	1	0	1	0.076	SLC45A2	Membrane-associated transporter protein
1	10	22	12	7	0.166	CNKSR2	Mediate the mitogen-activated protein kinase pathways
1	5	11	5	10	0.166	GPR68	G protein-coupled receptor 1, OGR1 responds to extracellular acidity and regulates a variety of cellular functions
1	8	1	0	12	0	IZUMO1R	Gamets adhesion
1	0.333	0.266	0.333	0.333	15	MYOZ2	Function in slow-twitch skeletal muscle, which functions to tether calcineurin to alpha-actinin at Z-discs
1	0.25	0.375	0.0625	0.125	16	TNNT2	Instructions for making a protein called cardiac troponin T
1	0.090	0.136	0.272	0	22	SFTPC	Production of surfactant protein-C

MRF demonstrated 2 structural genes *CIB3* (Integrin) and *CASS4* to have the highest fold change over the positive control and the other treatments (Table 3). Product A’s largest fold change increase relative to the positive control was for *NR4A3* which is important for cell growth and survival [29], MRF had the second highest fold change for this gene. Product B’s highest fold change increase over the positive control was for both *INHBA* required for cellular growth regulation [30] and *IGFBP4* also essential to stimulating or inhibiting growth [31]. Product C demonstrated an increased fold change over the positive control and other treatments for genes *TLL2* and *SGCA* both involved in the degradation and the formation of extracellular structures [32, 33]. The negative control had a mitochondrial adherence gene *ARMC12* with the largest fold change increase relative to the positive control and other treatments.

Overall MRF had three of its top five genes upregulated for cell structural genes, Product A had one, Product B with one and Product C with two genes. This result focused further assessment on genes associated with structural adhesion between cells.

### Differentially downregulated genes compared to the positive control

Genes with varied functions in neural signalling and maintenance (*CNTN5, GRID1, NPSR1* and *SV2B*) were reduced commonly in a number of treatments when compared to the positive control and negative control with the exception of MRFs *GRID1* and products A’s *SV2B* being elevated (Table 4). Transporter genes for calcium (*TRPM3*) and endogenous transporter *SLCO1A2* were lower in expression compared to the positive control. Interestingly the negative control had two genes *GPR4* and *NPSR1* that are involved in inflammatory regulation processes that were more elevated over the positive Control, MRF and products A, B and C.

**Table 4:** The top 5 downregulated gene transcription fold changes relative to the positive control for each treatment in response to LPS challenge or no challenge for the Negative control were compiled

Positive control	MRF	Product A	Product B	Product C	Negative control	gene_name	Description
1	0.166	0.166	0	0	6	ACR	Serine proteinase with trypsin-like specificity
1	0.142	0.571	0	0	7	CNTN5	Mediate cell surface interactions during nervous system development
1	0	0.428	0.285	0	7	CREG2	Roles in oxidoreductase activity and FMN binding
1	1	10	0	0	0.25	DOCK10	Dedicator of cytokinesis protein 10 involved in intracellular signalling networks
1	0	0	0.2	0.6	5	GPR4	A pro-inflammatory G protein-coupled receptor (GPCR) highly expressed in vascular endothelial cells
1	0	0	0	0	6	GATA5	Cooperative function in the activation of the intestinal lactase-phlorizin hydrolase promoter
1	6	0	3	1	0	GRID1	Glutamate receptor ligand-gated ion channel
1	0	2	5	0	0	LRRC14B	Leucine Rich Repeat Containing 14B
1	0	0	0	0	6	NPSR1	Membrane protein acts as a receptor for neuropeptide S and affects a variety of cellular processes and associated with inflammatory bowel disease
1	1	0	0	6	0	SLCO1A2	Mediates cellular uptake of a wide range of endogenous substrates
1	0	6	0	1	0	SV2B	control of regulated secretion in neural and endocrine cells
1	6	0	1	1	0	TDRD9	Required for transposon silencing in the nucleus
1	0	0.428	0	0	7	TRPM3	Non-selective calcium cation channel

### Gene ontology functional terms characterization analysis of differentially expressed genes between LPS challenged and treatment groups

**Fig. 3:**
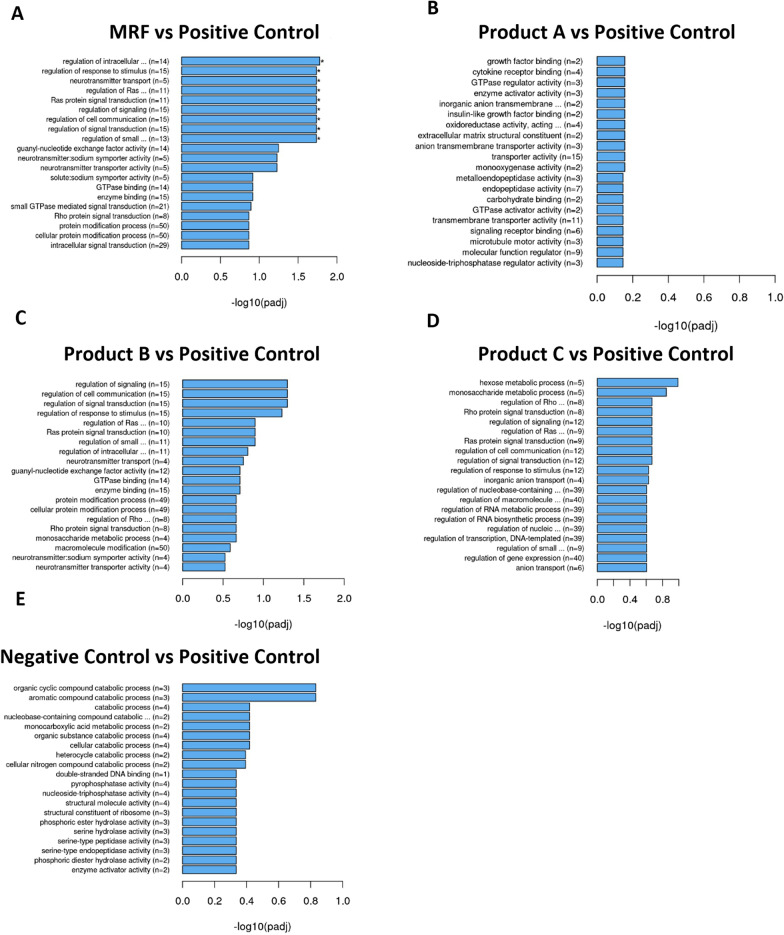
Gene ontology (GO) Terms grouped on functional classification of upregulated genes among positive control compared to MRF, Product A, B, C and Negative control groups. Gene Set Enrichment Analysis (GSEA) determines significant differences between two biological states. Significance marked with by *(*P* < 0.05) (*n* = 4)

Gene ontology results grouped based on function demonstrated significant differences between the positive control with MRF and products A, B and C. MRF highlighted significant (*P* < 0.05) increases in gene functions associated with ribosome function, peptide and amide processing, organelle activity, cell cycle and structural molecule activity (Fig. 3). Product A (Fig. 3B) demonstrated significant (*P* < 0.05) upregulation of genes associated to functions in microtubule arrangement and activity, enzymatic lead substrate breakdown and assembly and chromatin remodelling. Product B (Fig. 3C) demonstrated a number of significantly (*P* < 0.05) upregulated gene functions relative to the positive controls ribosome function, peptide and amide processing, enzymatic lead substrate breakdown, organelle activity and structural molecule activity.

Product C (Fig. 3D) highlighted similar trends to MRF and product B in gene functions that were significantly (*P* < 0.05) elevated over the positive control that included ribosome function, peptide and amide processing, organelle activity and structural molecule activity. Though product B had significantly more influence (*P* < 0.05) on upregulating gene functions associated with mitochondrion functions compared to the positive control IPEC-J2 cells. The negative control cells had significant (*P* < 0.05) upregulation in gene functions associated with ribosome function, peptide and amide processing, organelle activity and structural molecule activity over the positive control group.

**Fig. 4:**
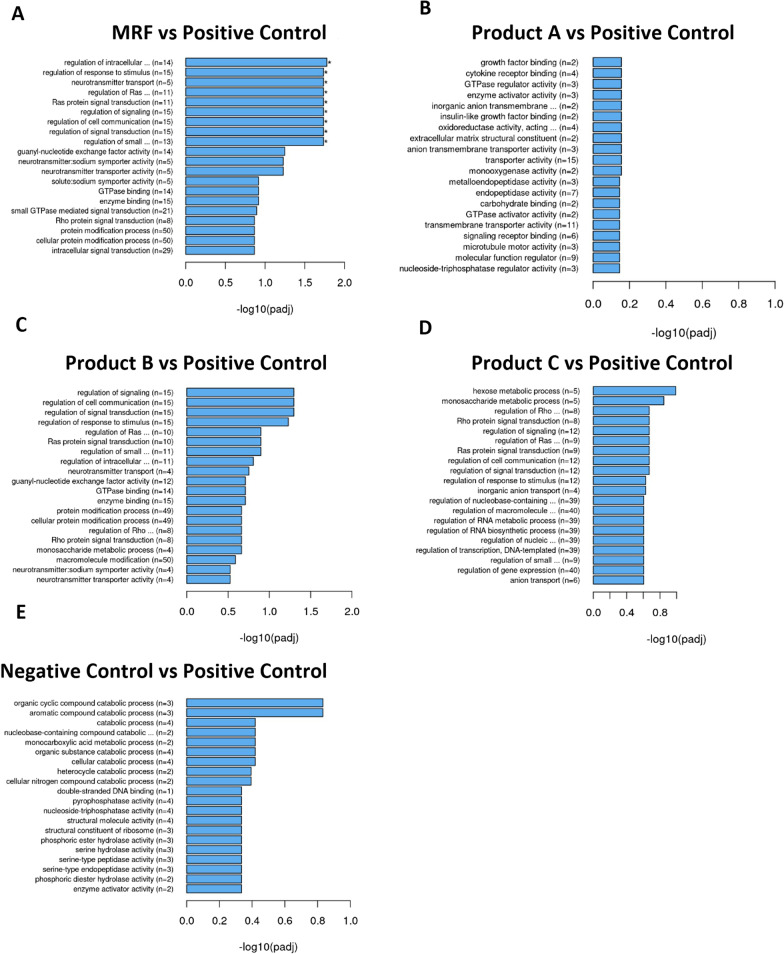
Gene ontology (GO) Terms grouped on functional classification of downregulated genes among positive control compared to MRF, Product A, B, C and Negative control groups. Gene Set Enrichment Analysis (GSEA) determines significant differences between two biological states. Significance marked with by *(*P* < 0.05) (*n* = 4)

MRF was the only treatment to demonstrate a significant (*P* < 0.05) downregulation of differentially expressed gene groups compared to the positive control (Fig. 4A). These genes were associated with molecule transport, cell signalling and receptor signalling processes that can regulate cell growth, proliferation, differentiation and immune responses.

### qPCR expression of junctional genes

**Fig. 5:**
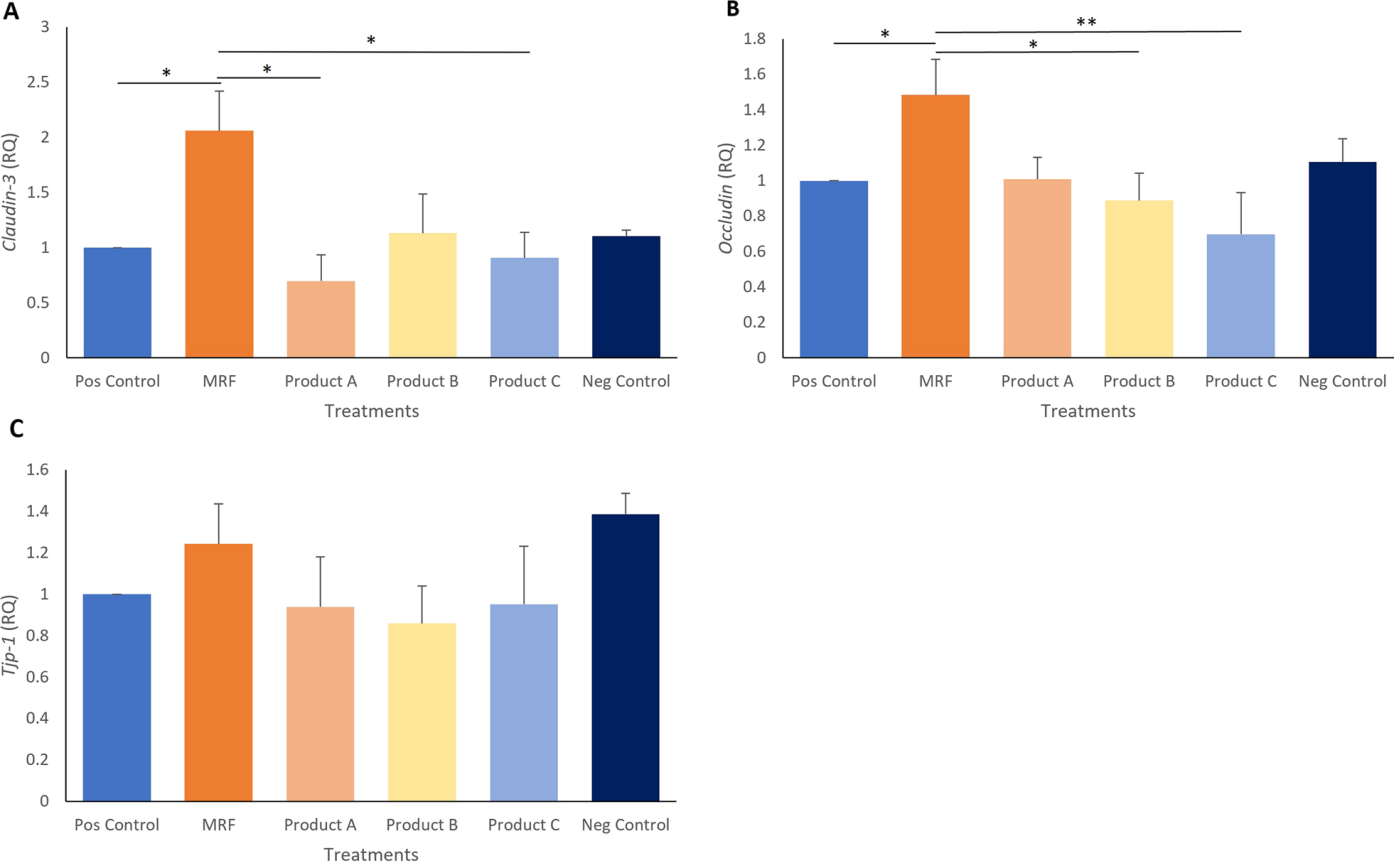
Gene expression of cell junction genes **A*** Claudin-3,* **B** *Occludin,* and **C** *TJP1* from differentiated IPEC intestinal cells challenged with *Salmonella* LPS and treated with MRF, product **A**, **B**, or **C** and positive Control (Pos Control) and negative Control (Neg Control). Gene expression levels were normalized to the reference group expression (*GAPDH*) and expressed as relative quantities (RQ). Significance marked with by *(*P* < 0.05) or **(*P* < 0.01) (*n* = 4)

Junctional gene expression for differentiated IPEC-J2 cells treated with MRF was significantly higher for *Claudin* 3 (2.06 ± 0.36, *P* ≤ 0.05) over the positive control (1 ± 0) and treatments A (0.69 ± 0.23, *P* ≤ 0.05) and C (0.91 ± 0.23, *P* ≤ 0.05) (Fig. 5A). *Occludin* expression was significantly higher in the MRF treated cells (1.48 ± 0.23, *P* ≤ 0.05) over the positive control (1.0 ± 0.0, *P* ≤ 0.05) and treatment B (0.88 ± 0.15, *P* ≤ 0.05) (Fig. 5B). *TJP-1* gene expression was highest in the MRF treated (1.248 ± 0.19) but not significantly higher than the positive control (1.0 ± 0.0) (Fig. 5C).

### Western blotting of junctional protein abundance

**Fig. 6:**
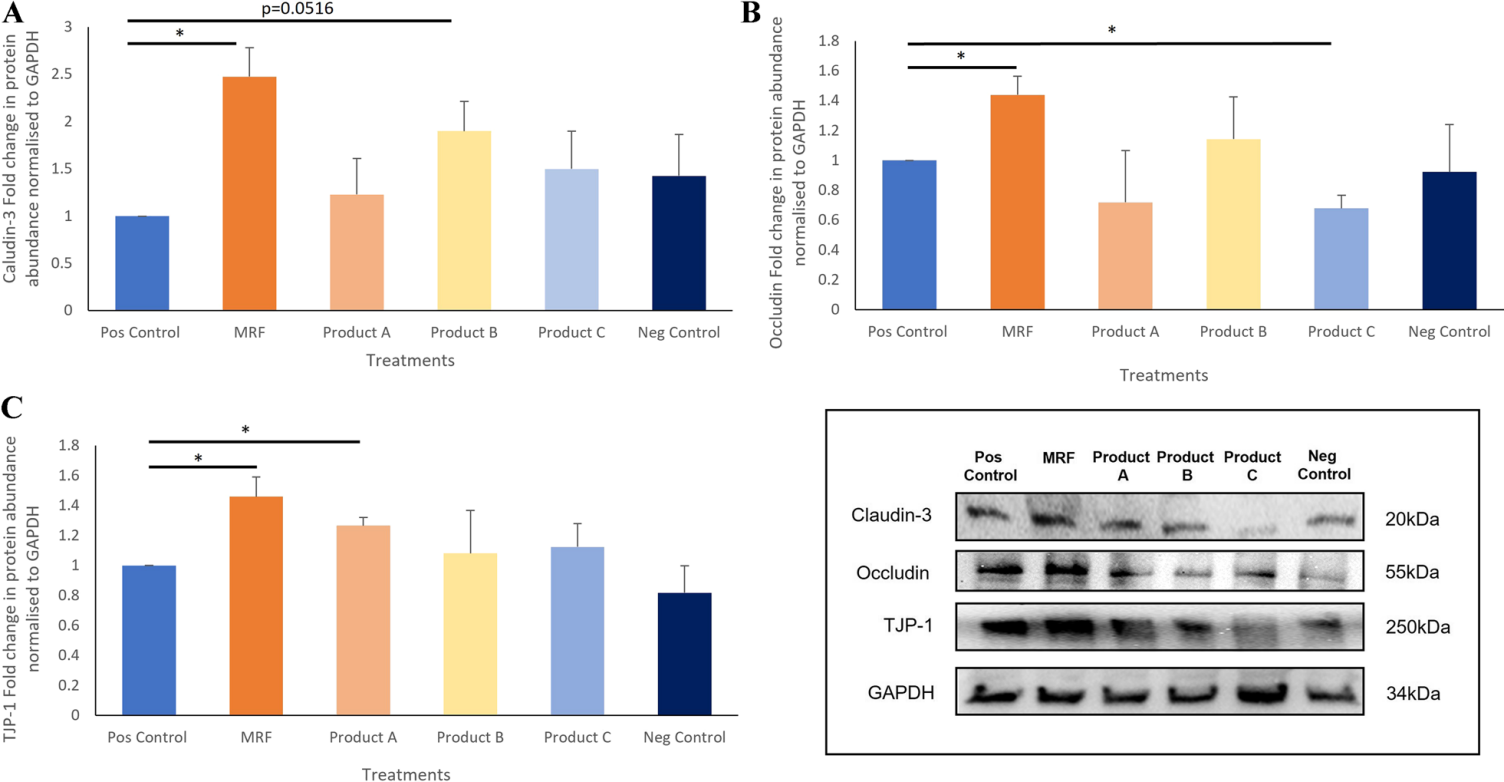
Western blotting of differentiated IPEC-J2 cell protein following LPS challenge and treatment with Yeast cell wall (YCW) products A, B C and MRF with a positive Control (Pos Control) and negative Control with no LPS (Neg Control). Target protein abundances **A** Claudin-3, **B** Occludin and **C** Tight junction protein 1 was normalised to GAPDH housekeeping protein abundance and western blotting representative images below respective graphs. Significance marked with by *(*P* < 0.05) (*n* = 4)

The abundance of Claudin-3 demonstrated a significant rise in differentiated IPEC-J2 cells receiving MRF over the positive control (Fig. 6A). These results correlate with the results observed from *Claudin 3* gene expression following MRF treatment in response to LPS challenge.

Occludin protein abundance post LPS challenge and MRF exposure demonstrated a significantly higher abundance of occludin (*P* ≤ 0.05) compared to the positive control (Fig. 6B). Whereas product C demonstrated a significant decrease (*P* ≤ 0.05) in occludin abundance compared to the positive control. Product A resulted in lower abundance comparable to the abundance of IPEC-J2 cells exposed to product C.

TJP-1 protein abundance was higher in IPEC-J2 cells exposed to YCW products although only two were significantly higher than the positive control (Fig. 6C). TJP-1 abundance was significantly higher in the IPEC-J2 cells supplemented with MRF (*P* ≤ 0.05) and Product A (*P* ≤ 0.05) over the positive control although MRF was slightly higher.

## Discussion

YCW usage in feed offers an alternative to antibiotics in minimising the impact of gram-negative bacteria that disrupt the intestinal tract of pigs. Due to the availability of multiple YCW products on the market this work’s emphasis was to determine differences in their effectiveness at impacting the barrier function of IPEC-J2 cells following LPS challenge. As a consequence of repeated microbial challenges and stresses to the intestinal tract of piglets [2, 9] it requires sustained support to improve the barrier function and reduce the number of microbial challenges that may cause harm to a growing pig.

The effects of YCW products post LPS challenge on IPEC-J2 cells highlighted significantly higher barrier function at recovery following MRF application compared to YCW products in comparison to the positive control (Fig. 1). Both Fouhse et al., [34] and Zhang et al., [35] demonstrated similar improvements in piglet’s intestinal tract development, function and villi height in response to MRF inclusion in feed. The differences in recovery between YCW products and MRF may result from variations in the strains of yeast used and the isolation process of yeast cell wall material which may impact product compositions and thus its effect on the barrier protection.

Interestingly Christie et al., [36] highlighted the rapid repair of epithelial cells when mannan was introduced to wounded epithelial cells that led to cellular ingrowth within a 32 h period which may be a factor in the enhanced TEER recovery results observed here following MRF application. Wang et al., [37] observed similar improvements in intestinal morphology and intestinal tight junction protein expression in pigs with the addition of a comparable yeast product following H_2_O_2_ injury with tight junctional proteins a key component to barrier recovery.

Taken together with epithelial barrier TEER recovery results following MRF addition, potentially highlights MRFs ability to help maintain barrier integrity which has important implications in enabling optimal nutrient absorption and protection from bacterial infection in pigs.

The application of LPS to IPEC-J2 cells as a simulated microbial challenge was validated from the presence of 3458 uniquely differentially expressed genes observed in the positive control receiving LPS compared to the negative controls 2 unique genes without LPS (Fig. 2A). This highlights the strong response IPEC-J2 cells have following LPS challenge at a molecular level.

Determining the impact of yeast cell wall products on the intestinal cells was further highlighted in RNA transcriptome analysis with functional classification of differentially expressed genes. Upregulated GO terms grouped on gene expression functions demonstrated a number of differentially expressed genes that were mainly involved in cellular maintenance. The GO term for structural molecule activity was demonstrated to be significantly upregulated in MRF treated cells challenged with LPS over the positive control with 56 genes upregulated following MRF application compared to product B with 50 genes, Product C’s 25 genes and the negative controls 60 genes although product A had no functional grouping under this term (Fig. 3). GO terminology determines Structural molecule activity as molecules that contribute to the structural integrity of a complex or assembly within or outside a cell such as the extracellular matrix. The result highlighted genes with structural functions were influenced following YCW applications compared to the positive control with MRF demonstrating the greatest number of these upregulated genes compared to products A, B and C and a comparable number to the negative control. Notably these genes are responsible for supporting barrier integrity and recovery. This result along with the table of top five transcriptome gene fold changes with roles in cell structure led to further focus on genes and proteins associated with cell structural and barrier maintenance functions. As previous findings of suckling piglets reared on sows receiving high levels of MRF demonstrated greater cell proliferation in the Jejunum which is important to facilitating intestinal tract maintenance following damage [38].

Three key junctional genes associated with barrier function *Occludin*, *Claudin3* and *TJP-1* were assessed by qPCR analysis with significantly higher junctional gene expression for *Claudin3* and *Occludin* observed in cells receiving MRF compared to the positive control during the TEER recovery phase (Fig. 5A and 5B). This likely contributed to the higher TEER readings from the IPEC-J2 intestinal cells exposed to MRF which is comparable to the improved intestinal integrity observed by Wang et al., [37]. Further assessment on protein abundance was determined on the three junctional genes via western blotting for which revealed a similar trend in abundances of Occludin, Claudin3 and TJP-1 to the qPCR expression (Fig. 6A, 6B and 6C) and RNA sequencing data (Additional file 1: Fig. S1). Tight junctions serve as a complex of proteins including Claudins, Occludin and TJP’s that are crucial to the maintenance of the intestinal barrier integrity [39]. With higher abundances of tight junction proteins observed here enabling the control of leakiness of the epithelium by regulating pore size and ion transition across the epithelium [1, 2, 37], that may facilitate a stronger barrier function in IPEC-J2 cells receiving MRF.

An impaired intestinal barrier is commonly seen in weanling piglets that leads to a leaky intestinal tract which can be triggered by post weaning stress that increases both inflammation and a dysbiosis of the intestinal microbiota in piglets contributing to greater bacterial invasion by pathogens [40, 41]. Despite many piglets overcoming the weaning stage and performing well, early intestinal trauma during weaning may alter the barrier strength that can infer future negative impacts on barrier function and susceptibility to infections up to slaughtering [2]. A leaky intestinal tract can lead to poor absorption of nutrients which could affect pig performance in weight and overall health. Many enteropathogenic bacteria have developed specific strategies to weaken the tight junction components to promote and facilitate their invasion [42, 43]. This increased risk of infection and colonisation by *Salmonella* and *E. coli* also increases the potential for foodborne diseases to be transmitted to the human consumer. A number of key factors affect YCW products such as S. *cerevisiae* yeast source and the manufacturing process of isolating the yeast cell wall which both may account for variations in performance on the IPEC-J2 cells barrier integrity following LPS challenge. The intestinal barrier acts as the first line of defence to microbial invasion, with the use of YCW products especially those rich in mannan may provide some benefit in maintaining a strong intestinal barrier and barrier recovery as seen here in vitro with MRF. MRF also may offer an effective alternative to improving barrier integrity of pigs in the absence of both antibiotics and therapeutic doses of zinc oxide.

## Material and methods

### Product reagent preparation

A mannan rich fraction was obtained from Alltech Inc., Nicholasville, Kentucky. An enzymatic digestion was performed on MRF and products A, B and C adapted from a 3-step in vitro technique by Boisen and Fernandez [27] with experimental volumes outlined by Meunier et al., [28]. Digests of MRF and yeast mannan products A, B and C were filtered in 10 kDa spin filters (Vivaspin® 20) to remove pepsin and pancreatin following adjustment to a final optimal protein concentration of 3 mg/ml (3000 ppm) of YCW products before application.

### Cell culture

Undifferentiated IPEC-J2 cells (DSMZ) were grown in media composed of DMEM high glucose (Gibco) with 10% v/v Foetal Bovine Serum (FBS), 1% v/v L-Glutamine added. Splitting was performed using Trypsin–EDTA.

### Trans epithelial electrical resistance

IPEC-J2 incubated in DMEM with 5% v/v Foetal Bovine Serum (FBS) and 1% v/v L-Glutamine, were trypsinised and resulting cell suspensions were diluted with the same media to 5 ml. Collagen coated Transwells (Corning, CLS3493) were added to 12-well plates and 150 μl differentiation media added and left to soak in an incubator. Differentiation media consisted of DMEM, 5% FBS, 2 mM L-Glutamine, 1 × Insulin-Transferrin Selenium (ITS), 5 ng/ml epidermal growth factor (EGF), 100 μg/ml penicillin and 100 μg/ml streptomycin.

Cells were counted manually with a haemocytometer and enough cell suspension added to give 2 × 10^5^ cells per well. Plates were labelled and incubated for several hours to allow attachment. 0.5 ml differentiation media was added to each trans well (this is apical media) and 1.5 ml differentiation media was added to the base wells (basal media) of the 12 well plate. Plates were sealed and incubated overnight in 37 °C, 5% CO_2_. Apical and basal media were changed every alternative day. TEER Readings were taken each day, recordings in Ohms (Ω) were recorded from each of the 3 read points of each well, giving 3 technical replicates spread over 4 biological replicates.

Differentiated IPEC-J2 cells grown to 14 days until a stable trans-epithelial electrical reading (TEER) of ~ 4500 Ohms/cm^2^ was reached. Prior to LPS (1 µg/mL) challenge, cells were pre-treated with MRF or YCW products A, B, and C (3 mg/mL) and incubated overnight. Wells were washed in warm HBSS, and cells were challenged with LPS (1 µg/mL in media) for 30 min and TEER readings were taken prior to washing cells and reapplying media with respective YCW products added. Cells were incubated overnight with the last TEER readings carried out 24 h post challenge at the recovery phase.

### Gene expression

Differentiated IPEC-J2 cells were lysed at the recovery phase in RTL buffer (Qiagen) scraped and ruptured for 30 s with a tissue rupture probe on ice to generate a homogenous mixture. RNA was isolated following the RNeasy Micro Kit (Qiagen, Germany) procedure as detailed in the user manual. RNA quantity integrity and quality determined by Qubit 4 Fluorometer (Invitrogen) with IQ values above 6.5 utilised and RNA concentrations ˃1.8 µg were used for both Novogene RNA sequencing and qPCR cDNA synthesis (SuperScript®-III).

### mRNA Sequencing

Transcriptome analysis by Novogene, identifies genes that are differentially expressed in distinct cell populations using high-throughput sequencing platform (Illumina) where data is transformed to sequenced reads (Raw Data or Raw Reads) by CASAVA base recognition (Base Calling). The raw data in the form of FASTQ(fq) format files are utilised for analysis following quality control.

Data quality control determines error rate and GC content distribution. The error rate for each base was transformed by the Phred score as in Eq. 1. "e" represents sequencing error rate, "Qphred" represents base quality values of Illumina platforms (Eq. 1: Qphred = -10log10(e)). GC content distribution detects potential AT/GC separation, which affects subsequent gene expression quantification. This follows removal of low quality reads or reads with adaptors, which will affect the quality of downstream analysis.

Analysis of differentially expressed genes by algorithm mapping of sequences using HISAT2, maps filtered sequence reads to the Sus Scrofa (Pig) genome. Mapped regions classified as exons, introns, or intergenic regions. With RNA-seq samples, gene expression level is estimated by the abundance of transcripts (count of sequencing) that mapped to the genome or exons. Read count is proportional to gene expression level, gene length and sequencing depth. FPKM (short for the expected number of Fragments Per Kilobase of transcript sequence per million base pairs sequenced) estimates gene expression levels, which takes the effects into consideration of both sequencing depth and gene length of counting fragments [44]. The false discovery rate (FDR) was corrected by Benjamini–Hochberg multiple tests, and transcripts with FDR < 0.05 were considered differentially expressed genes (DEGs). FPKM values were normalised to the positive control and fold change expressed on heat mapping software (Morpheus).

### GO Terminology analysis

RNAseq data enrichment analysis of the differentially expressed genes, were grouped on biological functions (Novogene). Shared functions among genes were incorporated by biological knowledge provided by biological ontologies. Gene Ontology (GO) annotates genes to biological processes, molecular functions, and cellular components in a directed acyclic graph structure, Kyoto Encyclopedia of Genes and Genomes. Gene Set Enrichment Analysis (GSEA) was used to determine whether the prior gene set was significantly different between two biological states (eg, phenotype).

### Validation of RNA-Seq expression profiles by RT-PCR

Real-time PCR was performed on four biological replicates using a two-step cycling programme, consisting of a heat activation step (95 °C for 10 min) and a cycling step (40 cycles, 95 °C for 15 s and 60 °C for 1 min) (ABI 7500 Fast; Applied Biosystems, USA). Junctional genes *Occludin*, *Claudin-3* and *Tight junction protein1* (*TJP1*), primers are listed in Table 1. Relative quantification (RQ) was determined by the delta delta Ct method.

### Protein analysis

Protein targets Occludin, Claudin 3 and TJP-1 ascertained from the gene expression changes of qPCR were assessed by Western blotting. Protein was isolated from differentiated IPEC-J2 cells at the recovery phase following washing of the cells and resuspending cells in RTL lysis buffer containing a protease inhibitor cocktail (Sigma Aldrich, P2714) and protein was quantified by Qubit™ Protein Assay Kit (Thermofisher, Q33211) and assay standard. Protein (50 μg) was loaded in each well of a 10–20% polyacrylamide gradient gel, run at 70–90 Volts for 15 min and 110 Volts until tracking dye reached the end of the gel. Polyacrylamide gels were transferred to a PVDF membrane (Millipore, IPVH15150) at 25 V for 45 min on a semi wet transfer sandwich (Thermo Scientific Pierce G2 Fast Blotter).

Resulting blots were ponceau stained to check for correct transfer of equal protein prior to blocking in blocking solution (1% BSA / 5% Milk protein resuspended in PBS Tween 20 (0.05%)) for 30 min. All blots were probed with target antibody separately in blocking solution for either Rabbit Anti-pig Claudin-3 (ABCAM, AB15102), Mouse Anti-pig Occludin (Invitrogen, OC-3F10) or Rabbit Anti-pig TJP-1 (Invitrogen, 61–7300) and for housekeeping protein GAPDH using Rabbit Anti-pig GAPDH (SIGMA, G9545). Each primary antibody was individually incubated for 1 h at room temperature (RT). Secondary Horseradish Peroxidase (HRP) linked antibody for Anti-rabbit-HRP (Abcam, AB191866) or Anti-mouse-HRP (Sigma, A9044) were probed for 1 h at RT to bind to the primary antibody and washed in PBS Tween 20 (0.05%) (3 × 10 min) for steps between antibody applications. Resulting Western blotting images were developed by SuperSignal™ enhanced chemiluminescence (ECL) substrate (Thermo scientific, 34,094) and analysed using the Invitrogen- Thermo scientific iBright FL1000 scanner.

### Statistical analysis

Four independent biological replicates were performed, with statistical analysis of TEER, western blots and gene expression experiments analysed by One-way ANOVA, Tukeys post-hoc test with significance determined at *P* ≤ 0.05, Graph Pad PRISM. Bar charts are represented as the mean average and standard error mean (SEM). Positive control was exposed to LPS only and the negative control contains only media no LPS or treatments.

## Supplementary Information

**Additional file 1.** **Junctional gene expression image and table:** **Fig. S1 A** Heat map and statistical significance **B** of differentiated IPEC-J2 intestinal cell junctional gene expression of MRF, products A, B, C, or the negative control compared to the positive control. Down regulated and upregulated genes relative to the positive control signified by arrows ↓ and ↑ respectively and significance marked with by *(*P* < 0.05) **(*P* < 0.01), *** (*P* < 0.001) (*n* = 4).

## Data Availability

All data will be available from the corresponding author upon reasonable request.

## Abbreviations

MRF: Mannan rich fraction
YCW: Yeast cell wall
TEER: Trans epithelial electrical resistance
TJP1: Tight junction protein 1
LPS: Lipopolysaccharide
IPEC-J2: Intestinal porcine epithelial cell, jejunum 2
