# Supplementary figures and images for: Differential impact of yeast cell wall products in recovery of porcine intestinal epithelial cell barrier function following Lipopolysaccharide challenge
Source: Porcine Health Manag. 2023 Apr 17;9:18. doi: 10.1186/s40813-023-00312-2 (PMC10111678; doi:10.1186/s40813-023-00312-2)

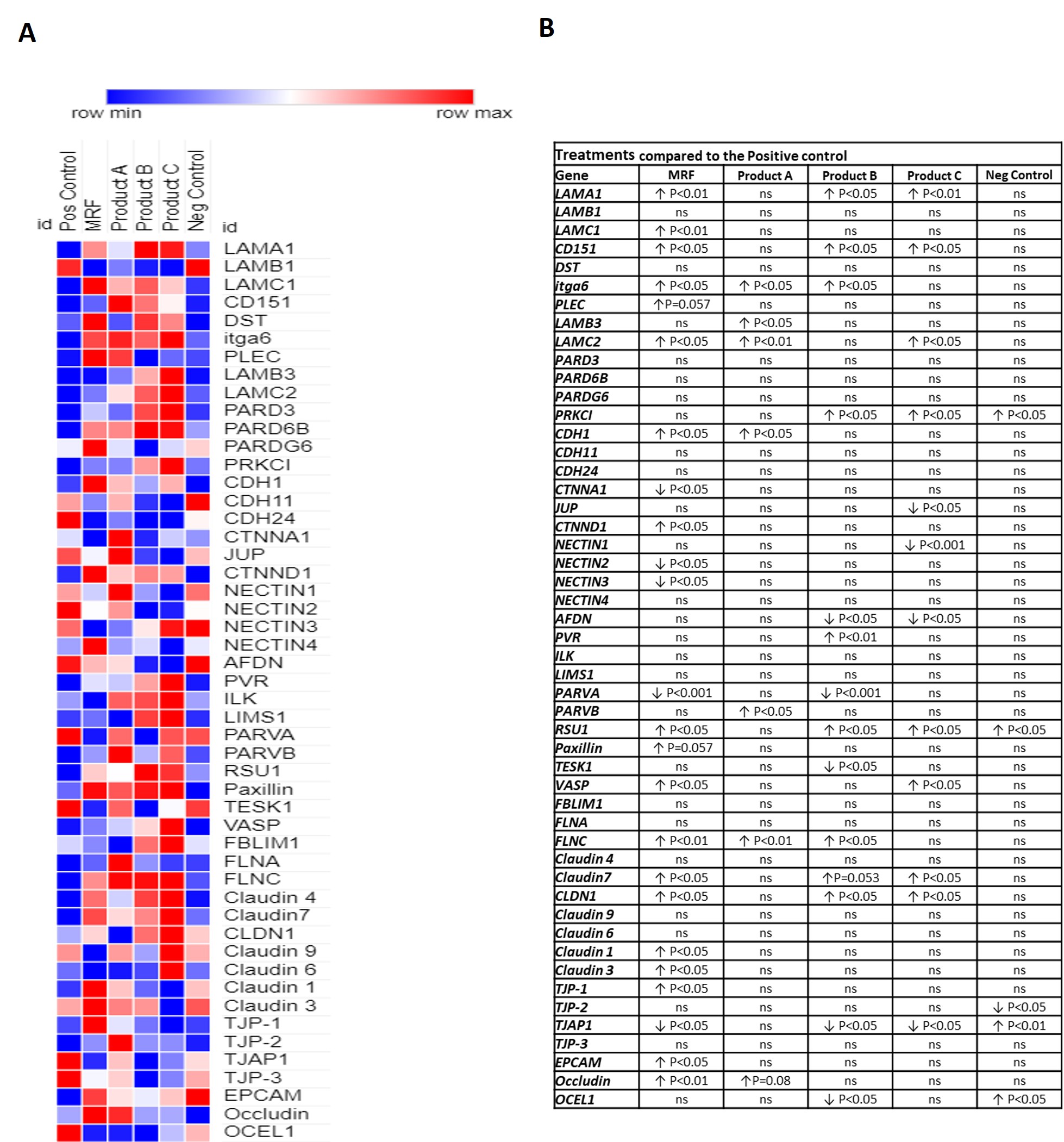

Supplement: Supplementary file 1 — Additional file 1. Junctional gene expression image and table: Fig. S1 A Heat map and statistical significance B of differentiated IPEC-J2 intestinal cell junctional gene expression of MRF, products A, B, C, or the negative control compared to the positive control. Down regulated and upregulated genes relative to the positive control signified by arrows ↓ and ↑ respectively and significance marked with by *(P < 0.05) **(P < 0.01), *** (P < 0.001) (n = 4). [file 40813_2023_312_MOESM1_ESM.jpg]
